# Reducing expectations for antibiotics in primary care: a randomised experiment to test the response to fear-based messages about antimicrobial resistance

**DOI:** 10.1186/s12916-020-01553-6

**Published:** 2020-04-23

**Authors:** Laurence S. J. Roope, Sarah Tonkin-Crine, Natalie Herd, Susan Michie, Koen B. Pouwels, Enrique Castro-Sanchez, Anna Sallis, Susan Hopkins, Julie V. Robotham, Derrick W. Crook, Tim Peto, Michele Peters, Christopher C. Butler, A. Sarah Walker, Sarah Wordsworth

**Affiliations:** 1 Health Economics Research Centre, Nuffield Department of Population Health, University of Oxford, Old Road Campus, Headington, Oxford, OX3 7LF UK; 2 The National Institute for Health Research Health Protection Research Unit in Healthcare Associated Infections and Antimicrobial Resistance at the University of Oxford, Oxford, UK; 3 NIHR Oxford Biomedical Research Centre, John Radcliffe Hospital, University of Oxford, Oxford, UK; 4 Nuffield Department of Primary Care Health Sciences, University of Oxford, Oxford, UK; 5 Centre for Behaviour Change, University College London, London, UK; 6 NIHR Health Protection Research Unit, Healthcare Associated Infection and Antimicrobial Resistance at Imperial College, London, UK; 7 Public Health England, London, UK; 8 Healthcare-Associated Infection and Antimicrobial Resistance Division, National Infection Service, Public Health England, London, UK; 9 Directorate of Infection, Royal Free London NHS Foundation Trust, London, UK; 10 Modelling and Economics Unit, National Infection Service, Public Health England, London, UK; 11 Nuffield Department of Medicine, University of Oxford, Oxford, UK; 12 Oxford University Hospitals NHS Trust, Oxford, UK; 13 Health Services Research Unit, Nuffield Department of Population Health, University of Oxford, Oxford, UK

**Keywords:** Inappropriate antibiotic use, Fear messages about antimicrobial resistance, Public campaigns

## Abstract

**Background:**

To reduce inappropriate antibiotic use, public health campaigns often provide fear-based information about antimicrobial resistance (AMR). Meta-analyses have found that fear-based campaigns in other contexts are likely to be ineffective unless respondents feel confident they can carry out the recommended behaviour (‘self-efficacy’). This study aimed to test the likely impact of fear-based messages, with and without empowering self-efficacy elements, on patient consultations/antibiotic requests for influenza-like illnesses, using a randomised design.

**Methods:**

We hypothesised that fear-based messages containing empowering information about self-management without antibiotics would be more effective than fear alone, particularly in a pre-specified subgroup with low AMR awareness. Four thousand respondents from an online panel, representative of UK adults, were randomised to receive three different messages about antibiotic use and AMR, designed to induce fear about AMR to varying degrees. Two messages (one ‘strong-fear’, one ‘mild-fear’) also contained empowering information regarding influenza-like symptoms being easily self-managed without antibiotics. The main outcome measures were self-reported effect of information on likelihood of visiting a doctor and requesting antibiotics, for influenza-like illness, analysed separately according to whether or not the AMR information was ‘very/somewhat new’ to respondents, pre-specified based on a previous (non-randomised) survey.

**Results:**

The ‘fear-only’ message was ‘very/somewhat new’ to 285/1000 (28.5%) respondents, ‘mild-fear-plus-empowerment’ to 336/1500 (22.4%), and ‘strong-fear-plus-empowerment’ to 388/1500 (25.9%) (*p* = 0.002). Of those for whom the respective information was ‘very/somewhat new’, only those given the ‘strong-fear-plus-empowerment’ message said they would be less likely to request antibiotics if they visited a doctor for an influenza-like illness (*p* < 0.0001; 182/388 (46.9%) ‘much less likely’/‘less likely’, versus 116/336 (34.5%) with ‘mild-fear-plus-empowerment’ versus 85/285 (29.8%) with ‘fear-alone’). Those for whom the respective information was not ‘very/somewhat new’ said they would be less likely to request antibiotics for influenza-like illness (*p* < 0.0001) across all messages (interaction *p* < 0.0001 versus ‘very/somewhat new’ subgroup). The three messages had analogous self-reported effects on likelihood of visiting a doctor and in subgroups defined by believing antibiotics would ‘definitely/probably’ help an influenza-like illness. Results were reproduced in an independent randomised survey (additional 4000 adults).

**Conclusions:**

Fear could be effective in public campaigns to reduce inappropriate antibiotic use, but should be combined with messages empowering patients to self-manage symptoms effectively without antibiotics.

## Background

Antimicrobial resistance (AMR) is an increasingly serious threat to global public health [1], estimated to account for 10 million deaths annually worldwide by 2050, and potentially imposing similar economic costs as climate change [2, 3]. A recent European study found that the burden of antibiotic-resistant infections (excluding tuberculosis) in terms of disability-adjusted life-years (DALYs) is similar to influenza, tuberculosis, and HIV combined [4]. Strong links have been found between the volume of human antibiotic consumption and AMR [5–9]. Thus, better stewardship of existing antibiotics, reducing inappropriate use without compromising essential access, is crucial to control AMR [3, 10–13].

Respiratory tract infections (RTIs) account for around 50% of antibiotic prescribing in primary care [14], much of which fails to benefit patients as the infections are self-limiting or viral [15]. Antibiotic use for RTIs not only affects resistance among possible causative bacterial pathogens, but also drives resistance among commensal flora, which can later cause resistant infections [9]*.*

Differences in clinical consultation rates explain much of the variation in non-hospital antibiotic usage in England [16], and clinicians are more likely to prescribe antibiotics when patients request them or are perceived to want them [17–19]. Thus, in healthcare systems such as that of the United Kingdom (UK), where general practitioners act as gatekeepers to prescriptions, decisions whether to consult, and whether to request antibiotics, for RTIs could each play a significant role in ‘unnecessary’ antibiotic consumption. A review of public campaigns [20] aimed at improving antibiotic use among outpatients in high-income countries concluded that several campaigns had a positive effect. However, nearly all were multi-faceted, simultaneously targeting physicians and the public with multiple interventions. Therefore, it was difficult to unpick whether improvements were due to changes in the behaviour of patients, physicians, or both or whether the observed improvements were related to confounding factors which would have happened without the campaigns. All campaigns in the review tried to convey the message that AMR is a serious problem, sometimes using a fear message. Most also tried to educate the public that antibiotics are often ineffective for RTIs.

However, in other healthcare areas, meta-analyses have found that while such ‘fear-appeal’ messages can be effective, they are likely to be ineffective and may even backfire if people are not confident that they will be able to successfully take the recommended action (‘self-efficacy’) or that the recommended action will be effective (‘response-efficacy’) [21–23]. In a previous survey [24], we found that an AMR ‘fear message’, which contained no information about how to successfully manage influenza-like illness (ILI) symptoms (generally not requiring antibiotics), was likely to backfire among those with low AMR awareness: paradoxically, significantly more of these respondents said they would be more likely, rather than less likely, to ask a doctor for antibiotics for ILI given the information. We hypothesised that fear-based messages also containing empowering information about successful self-management without antibiotics would be more effective, particularly among those with low AMR awareness, who we hypothesised would respond less favourably than others to fear-based messages in general. We therefore developed an experiment to test several AMR ‘fear messages’, with and without empowering content intended to provide confidence that ILI symptoms can be successfully self-managed without antibiotics. The aim was to identify the form of words most likely to discourage people from asking for antibiotics for ILI, particularly among those with low AMR awareness (pre-specified based on our previous survey) and those who believe antibiotics are effective for ILI (exploratory analysis).

## Methods

The randomised experiment was conducted using an online survey of adult members of the UK general public, in two waves. Wave 2 was conducted as a validation study, using identical methods but analysed separately on an independent sample, after analysing data from wave 1.

### Survey design

The survey (Additional file 1) asked respondents to imagine Health State A: ‘a temperature, aching muscles, a headache, a dry chesty cough, a sore throat, and you feel weak’, representing, and hereafter referred to as, ILI. A symptomatic description was used, rather than specifying ‘flu’ or a ‘virus’, firstly, because individuals are likely to interpret specific terms differently, and secondly, because people may know that antibiotics are not indicated for viral conditions, yet not recognise ILI symptoms as being more consistent with a viral, than bacterial, infection.

Respondents were randomised to receive one of three different messages about antibiotics and AMR (Table 1). In each wave, *N* = 1000 received version 1, a ‘fear message’ about the dangers of AMR, and AMR being caused by inappropriate antibiotic use. Two other groups (both *N* = 1500 in each wave) also received a ‘fear message’ but together with empowering information that ILI symptoms can easily and effectively be self-managed without antibiotics, differing only by the strength of the ‘fear message’ (stronger fear level in version 3 than the relatively mild version 2). Version 1 was a slight variant of the ‘fear message’ previously found [24] to backfire among respondents who found this AMR information surprising. Version 2 and version 3 were developed based on health-psychological theory, incorporating empowering ‘self-efficacy’ and ‘response-efficacy’ aspects to mitigate or remove backfiring potential.

**Table 1:** Three versions of information about antibiotics and AMR given to survey respondents

**Survey version 1**	
Message summary	‘**Fear**’ message only
Sample size in each wave	*N* = 1000
Full text	‘Antibiotic resistance happens when an antibiotic no longer kills or controls growing bacteria. It is an increasingly serious threat to public health. Without antibiotics that work well, many routine treatments will become increasingly dangerous. Setting broken bones, and even basic operations, rely on access to antibiotics that work. Antibiotic resistance is believed to be caused by unnecessary use of antibiotics, and inappropriate use, such as not taking them as prescribed, skipping doses, or saving them for later use.’
**Survey version 2**	
Message summary	‘**Mild fear**’ *plus****empowerment***
Sample size in each wave	*N* = 1500
Full text	‘Most people get cold or flu symptoms every year, and these usually get better on their own. Temperatures sometimes last for days, while coughs can last for weeks, and antibiotics generally don't help. Antibiotics should not be taken for cold and flu symptoms. Taking antibiotics when they are not needed encourages bacteria to become resistant. This means antibiotics may not work for future serious illnesses that can only be cured by antibiotics. Most cold and flu symptoms are best treated at home by taking paracetamol or ibuprofen, and getting plenty of fluids and sleep.’
**Survey version 3**	
Message summary	‘**Strong fear**’ *plus****empowerment***
Sample size in each wave	*N* = 1500
Full text	‘Most people get cold or flu symptoms every year, and these usually get better on their own. Temperatures sometimes last for days, while coughs can last for weeks, and antibiotics generally don't help. Antibiotics should not be taken for cold and flu symptoms. Taking antibiotics encourages bacteria to become resistant. Some killer diseases are already resistant to several antibiotics. Antibiotic resistance is an increasingly serious threat to everyone’s health. Soon we will not be able to find antibiotics that can cure serious illnesses. Even worse, without antibiotics that work, even minor injuries and routine operations will become increasingly dangerous. You can also pass on resistant bugs to people you care about. Most cold and flu symptoms are best treated at home by taking paracetamol or ibuprofen, and getting plenty of fluids and sleep.’

Respondents were then asked ‘To what extent is this information new to you?’ [very new/somewhat new/not very new/not at all new], ‘How will this information affect whether you visit a doctor the next time you have symptoms like Health State A?’ [much more likely to visit/more likely to visit/no effect/less likely to visit/much less likely to visit/do not know], and ‘How will this information affect the likelihood of you asking a doctor for antibiotics if you were to visit for these symptoms?’ [much more likely/more likely/no effect/less likely/much less likely/do not know] (co-primary outcomes: *likelihood of visiting a doctor and of requesting antibiotics*). Respondents with children were additionally asked analogous questions regarding how they would act if their youngest child had ILI symptoms (secondary outcomes in the subgroup with children).

Based on our previous survey [24], the impact of each randomised message on all outcomes was considered separately by whether or not respondents said the AMR information was ‘very/somewhat new’, formally compared using interaction tests. A limitation of comparing respondents for whom information is ‘new’ across the three versions is that this may not compare like with like—for example, some respondents might find version 3 ‘new’ but not version 2. In exploratory analysis, the impacts of the randomised messages were therefore also considered separately by whether or not respondents thought antibiotics would ‘definitely/probably help’ an ILI which persisted for 5 days.

The survey captured information on socio-demographic characteristics including age, sex, ethnicity, education, employment status, geographic region, and having dependent children. Both the original AMR message (‘fear-only’) and the survey were designed with clinical experts (*n* = 3; primary care physician and two junior doctors) and a public and patient involvement panel (*n* = 7). Amendments to the AMR message were designed primarily by health psychologist authors on this paper (STC, NH, SM, AS).

### Survey respondents

Both survey waves were conducted online using respondent panels provided by Survey Sampling International (SSI). In each wave, SSI was commissioned to obtain 4000 completed responses, representative of the UK adult population in terms of sex, age, ethnicity, and geographic region. Surveys were completed over 17 days during October/November 2016 (wave 1) and 22 days during March 2017 (wave 2). In wave 1, invitations were emailed to 28,887 SSI panel members; 8317 panel members were emailed in wave 2. It was also possible for SSI panel members to access the survey via SSI’s website. The two waves were completely independent, with no respondent overlap. Respondents were offered an incentive to complete the questionnaire in the form of ‘Nectar-points’ (a loyalty card scheme via which customers accrue discounts at outlets including supermarkets and restaurants), worth a total of approximately £0.60. To mitigate self-selection bias, specific project details were not included in the invitation. In each wave, sample sizes for groups receiving version 2 and version 3 were powered to quantify the proportion of individuals who, in response to the information, said they would be ‘less/much less likely’ to visit a doctor the next time they have ILI symptoms, at a 95% confidence level, with a maximum margin of error of 2.5%.

### Statistical analysis

Wilcoxon’s single-sample sign-rank tests were performed to test whether respondents were more or less likely to consult/request antibiotics for ILI in response to the information, overall and within subgroups defined by whether respondents said the information they were given about AMR was ‘very/somewhat new’ (and, in exploratory analysis, by whether antibiotics would ‘definitely/probably help’ an ILI). For each randomised message, Wilcoxon’s rank-sum tests were used to analyse whether the distribution of responses differed significantly between subgroups. Ordered logit regressions were used to test for interactions between message treatment effects and subgroups, with the extent of being more/less likely to consult/request antibiotics the dependent variables. All statistical analysis was conducted using Stata MP v13.1.

## Results

**Fig. 1:**
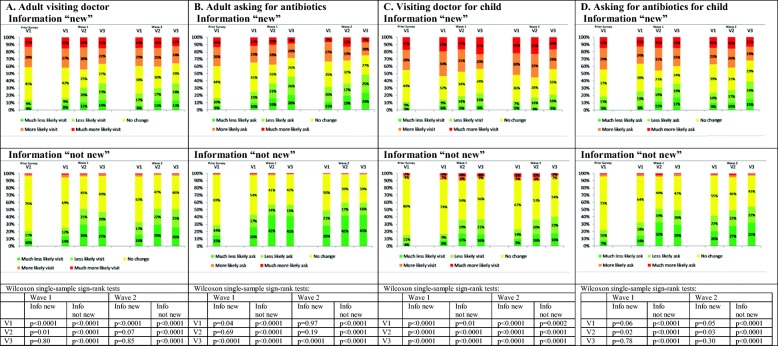
Reported impact of information on consultations/antibiotic requests disaggregated according to whether AMR information ‘new’ vs ‘not new’. (i) Prior Survey results are for May–June 2015 data reported in Roope et al. [24]; wave 1 conducted in October–November 2016; wave 2 conducted in March 2017. (ii) V1 is ‘fear-only’ message; V2 is ‘mild-fear-plus-empowerment’; V3 is ‘strong-fear-plus-empowerment’. (iii) Column charts exclude respondents who answered ‘do not know’ (< 8%). (iv) *p* values are from Wilcoxon’s single-sample sign-rank tests of whether respondents were more/less likely to consult/request antibiotics for ILI in response to the information they were given, under the null hypothesis that they were neither more likely nor less likely to consult/request antibiotics

Four thousand individuals completed the survey in October/November 2016, and another 4000 in March 2017. Baseline characteristics were broadly balanced across randomised messages (Table 2, Additional file 2: Table S1). As the two waves gave qualitatively very similar results (Fig. 1, details in Additional file 2: Tables S2, S3), we focus on the more-recent wave 2.

**Table 2:** Respondent characteristics in wave 2 (March 2017)

Factor	Version 1 (*N* = 1000): ‘fear-only’		Version 2 (*N* = 1500): ‘mild-fear-plus-empowerment’		Version 3 (*N* = 1500): ‘strong-fear-plus-empowerment’	
	Mean or number	Standard deviation or %	Mean or number	Standard deviation or %	Mean or number	Standard deviation or %
Age	47.2	16.7	46.5	16.8	46.0	16.7
Household equivalent income (£)^1^	20,571	14,431	20,164	15,107	20,166	15,379
Own self-rated health (0–10)	7.2	2.0	7.3	2.0	7.3	2.0
Male^1^	502	50.3%	724	48.3%	715	47.7%
White^1^	899	90.3%	1352	90.6%	1355	91.0%
Christian^1^	486	49.6%	700	48.0%	672	45.7%
Higher education	448	44.8%	658	43.9%	650	43.3%
Unemployed	41	4.1%	74	4.9%	69	4.6%
Sick or disabled	38	3.8%	64	4.3%	60	4.0%
Married/civil partnership/live with partner	674	67.4%	1015	67.7%	979	65.3%
Born in UK	906	90.6%	1351	90.1%	1347	89.8%
Geographic region						
East Anglia	90	9.0%	138	9.2%	144	9.6%
East Midlands	62	6.2%	111	7.4%	117	7.8%
West Midlands	88	8.8%	121	8.1%	145	9.7%
London	129	12.9%	198	13.2%	189	12.6%
North East	42	4.2%	55	3.7%	7.1	4.7%
North West	116	11.6%	177	11.8%	157	10.5%
South East	149	14.9%	196	13.1%	206	13.7%
South West	96	9.6%	120	8.0%	127	8.5%
Yorkshire and Humberside	81	8.1%	121	8.1%	135	9.0%
Wales	46	4.6%	88	5.9%	63	4.2%
Scotland	77	7.7%	132	8.8%	109	7.3%
Northern Ireland	24	2.4%	43	2.9%	37	2.5%
AMR information is ‘very/somewhat new’	285	28.5%	336	22.4%	388	25.9%
Antibiotics would ‘definitely/probably’ help ILI	279	27.9%	415	27.7%	418	27.9%
Antibiotics would ‘definitely/probably’ help child’s ILI	183/408	44.9%	255/587	43.4%	250/605	41.3%

^1^Version 1 denominator is 1000 except for household income (*N* = 940), gender (*N* = 999), ethnicity (*N* = 996), and religion (*N* = 979) where there was a ‘prefer not to answer’ option; version 2 denominator is 1500 except for household income (*N* = 1360), gender (*N* = 1499), ethnicity (*N* = 1493), and religion (*N* = 1459) where there was a ‘prefer not to answer’ option; version 3 denominator is 1500 except for household income (*N* = 1389), gender (*N* = 1498), ethnicity (*N* = 1489), and religion (*N* = 1469) where there was a ‘prefer not to answer’ option

### Reported response to information for respondents’ own ILI

In wave 2, overall, 29.2%, 45.1%, and 46.1% of respondents randomised to ‘fear-only’, ‘mild-fear-plus-empowerment’, and ‘strong-fear-plus-empowerment’ respectively reported that they would be ‘less/much less likely’ to visit a doctor as a result of the information given (*p* < 0.0001), while 14.1%, 10.3%, and 11.3% reported that they would be ‘more/much more likely’ to visit a doctor (*p* = 0.01) (Additional file 2: Table S3). For requesting antibiotics, 42.3%, 52.5%, and 54.7% respectively reported that they would be ‘less/much less likely’ to ask for antibiotics as a result of the information (*p* < 0.0001), while 10.1%, 8.2%, and 7.5% respectively reported that they would be ‘more/much more likely’ to ask for antibiotics (*p* = 0.08) (Additional file 2: Table S3).

**Fig. 2:**
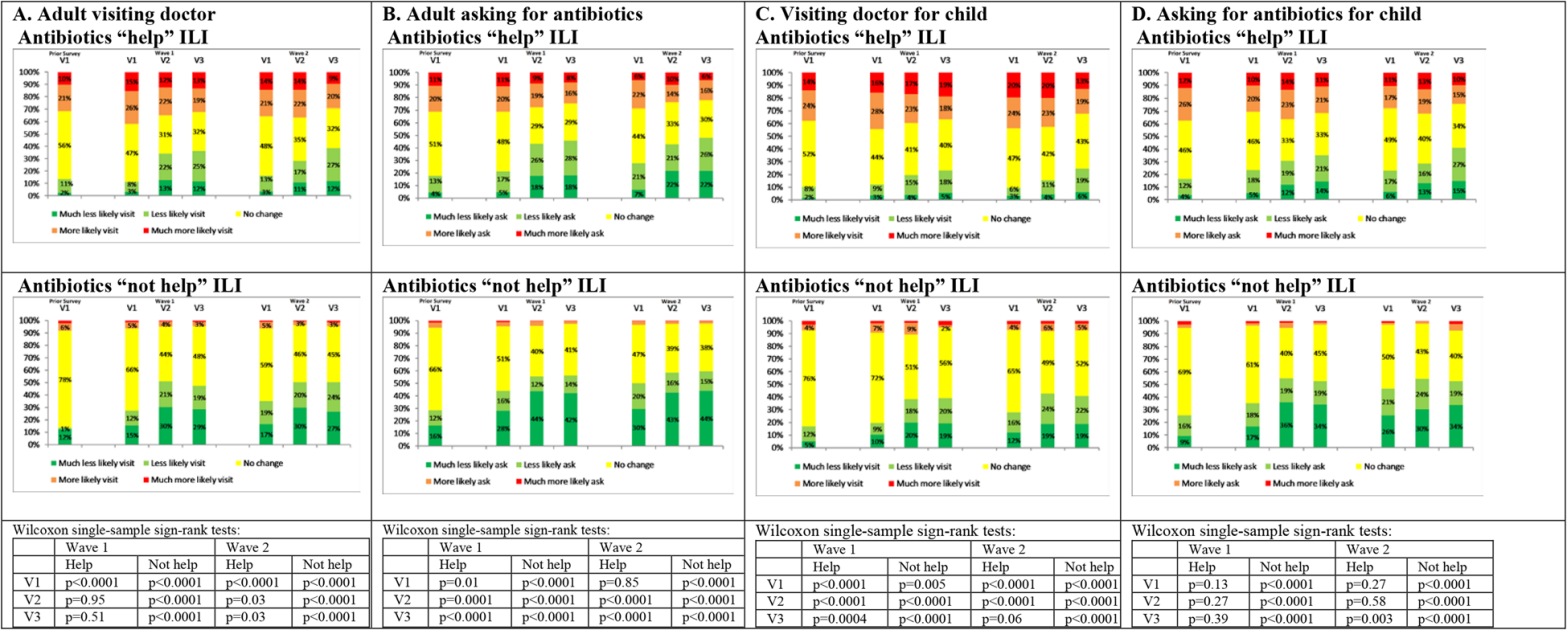
Reported impact of information on consultations/antibiotic requests disaggregated by whether or not antibiotics would ‘help’ ILI. (i) Prior Survey results are for May–June 2015 data reported in Roope et al. [24]; wave 1 conducted in October–November 2016; wave 2 conducted in March 2017. (ii) V1 is ‘fear-only’ message; V2 is ‘mild-fear-plus-empowerment’; V3 is ‘strong-fear-plus-empowerment’. (iii) Column charts exclude respondents who answered ‘do not know’ (< 7%). (iv) *p* values are from Wilcoxon’s single-sample sign-rank tests of whether respondents were more/less likely to consult/request antibiotics for ILI in response to the information they were given, under the null hypothesis that they were neither more likely nor less likely to consult/request antibiotics

Overall, 25.2% of respondents said the information they were randomised to was ‘very/somewhat new’, with small but significant variation between messages (*p* = 0.002, Table 2). Furthermore, 27.8% believed antibiotics would ‘definitely/probably’ help an ILI which persisted for 5 days, with no evidence of variation between messages (*p* = 0.99, Table 2). As hypothesised, respondents who found the information ‘very/somewhat new’ were less likely than the others to report that it would make them less likely to visit their doctor or ask for antibiotics (both ‘visit’ and ‘ask’ interactions *p* < 0.0001; Fig. 1a, b; Additional file 2: Table S3). Similarly, respondents who thought antibiotics would ‘definitely/probably’ help ILI were less likely than the others to report that it would make them less likely to visit a doctor or ask for antibiotics (both ‘visit’ and ‘ask’ interactions *p* < 0.0001; Fig. 2a, b; Additional file 2: Table S4; similar results for wave 1, Additional file 2: Table S5).

### Response to information for respondents’ own ILI: information not ‘very/somewhat new’

Among those for whom the information was not ‘very/somewhat new’, significantly more respondents said they would be less likely (rather than more likely) to consult/request antibiotics for ILI in response to each message (*p* < 0.0001; Fig. 1a, b), with < 5% reporting that they would be more likely to visit a doctor/request antibiotics regardless of message. Similarly, of those who did not think antibiotics would help ILI, significantly more said they would be less likely (rather than more likely) to consult/request antibiotics for ILI in response to each message (*p* < 0.0001; Fig. 2a, b).

### Response to information for respondents’ own ILI: information ‘very/somewhat new’

In contrast to respondents for whom the information was not ‘very/somewhat new’, among those for whom the information was ‘very/somewhat new’, and exposed to the ‘fear-only’ message, significantly more said they were more likely (rather than less likely) to consult for ILI (38.2% more likely versus 21.8% less likely; *p* = 0.0001, Fig. 1a). There was no significant difference between the proportion who said they would be more likely (versus less likely) to request antibiotics (*p* = 0.97; Fig. 1b). The results were similar for respondents exposed to the ‘fear-only’ message who believed antibiotics would ‘definitely/probably help’ (Fig. 2a, b), with 34.8% saying they were more likely, versus 16.1% less likely, to consult for ILI (*p* < 0.0001), and no significant difference between the proportions who said they would be more likely (versus less likely) to request antibiotics (*p* = 0.85).

However, those exposed to the ‘mild-fear-plus-empowerment’ message and who found the message ‘very/somewhat new’ said they were no more/less likely to consult for ILI (38.4% more likely versus 28.9% less likely; *p* = 0.07) and no more/less likely to request antibiotics (30.7% more likely versus 34.5% less likely; *p* = 0.19) (Fig. 1a, b). Of respondents who believed antibiotics would ‘definitely/probably help’ and exposed to the ‘mild-fear-plus-empowerment’ message, significantly more said they were more likely (rather than less likely) to consult for ILI (149 (35.9%) more versus 114 (27.5%) less; *p* = 0.03), but significantly more said they were less likely (rather than more likely) to request antibiotics (173 (41.7%) less versus 97 (23.4%) more; *p* < 0.0001) (Fig. 2a, b).

The differences between responses to the ‘fear-only’ and ‘strong-fear-plus-empowerment’ messages were even more pronounced than the differences between the ‘fear-only’ and ‘mild-fear-plus-empowerment’ messages. Among those who found the ‘strong-fear-plus-empowerment’ message ‘very/somewhat new’, there was no significant difference between the proportions who said they would be more likely (versus less likely) to consult (*p* = 0.85) and they were significantly less likely (rather than more likely) to request antibiotics (182 (46.9%) less versus 95 (24.5%) more; *p* < 0.0001) (Fig. 1a, b). Of respondents who believed antibiotics would ‘definitely/probably help’ and exposed to the ‘strong-fear-plus-empowerment’ message, significantly more said they were less likely (rather than more likely) both to consult (159 (38.0%) less versus 121 (28.9%) more; *p* = 0.03) and to request antibiotics (197 (47.1%) less versus 91 (21.8%) more; *p* < 0.0001) (Fig. 2a, b).

### Reported response to information for respondents’ children’s ILI

Overall, 496 (31.0%) of 1600 parents said the information they were randomised to was ‘very/somewhat new’, with no evidence of variation between messages (134/408 (32.8%) ‘fear-only’, 169/587 (28.8%) ‘mild-fear-plus-empowerment’, 193/605 (31.9%) ‘strong-fear-plus-empowerment’; *p* = 0.32, Additional file 2: Table S3). Furthermore, 688 (43.0%) of parents believed antibiotics would ‘definitely/probably’ help their child if they had an ILI which persisted for 5 days, with no evidence of variation across messages (*p* = 0.52, Table 2). As above, and as hypothesised, the outcomes depended strongly on whether or not respondents found the information provided about AMR ‘very/somewhat new’ (both ‘visit doctor’ and ‘ask for antibiotics’ interactions *p* < 0.0001) or said they thought antibiotics would ‘definitely/probably’ help ILI (both ‘visit doctor’ and ‘ask for antibiotics’ interactions *p* < 0.0001).

Overall results (Figs. 1c, d and 2c, d) were congruent with those for respondents themselves (details in Additional file 3). In brief, among parents for whom the information was not ‘very/somewhat new’ and who did not think antibiotics would help their child’s ILI, significantly more respondents said they would be less likely (rather than more likely) to consult/request antibiotics for their child’s ILI in response to each message (*p* < 0.001, Figs. 1c, d and 2c, d).

There were contrasting results among parents for whom the information was ‘very/somewhat new’. Of parents exposed to the ‘fear-only’ information, significantly more of those for whom the information was ‘very/somewhat new’ said they were more likely (rather than less likely) to consult for their child’s ILI and request antibiotics (*p* < 0.05) (Fig. 1c, d). Among parents exposed to this information who believed antibiotics would ‘definitely/probably’ help their child, significantly more said they were more likely (rather than less likely) to consult for their child’s ILI (*p* < 0.0001) (Fig. 2c, d), with no change in reported likelihood of requesting antibiotics (*p* = 0.27). Among parents receiving the ‘mild-fear-plus-empowerment’ message, significantly more of those for whom the information was ‘very/somewhat new’ said they were more likely (rather than less likely) to consult for their child’s ILI and request antibiotics (*p* < 0.03). Among parents exposed to this information who thought antibiotics would ‘definitely/probably’ help, significantly more said they were more likely (rather than less likely) to consult for their child’s ILI (*p* < 0.0001), with no change in reported likelihood of requesting antibiotics (*p* = 0.58). Among parents given the ‘strong-fear-plus-empowerment’ message, significantly more of those for whom the information was ‘very/somewhat new’ said they were more likely (rather than less likely) to consult for their child’s ILI (*p* < 0.0001), but in contrast to the other two randomised messages, they were not more likely (versus less likely) to request antibiotics for their child (31.1% more likely versus 37.8% less likely; *p* = 0.30). Moreover, parents given this message who thought antibiotics would ‘definitely/probably’ help were not significantly more likely (versus less likely) to consult for their child’s ILI (31.6% more likely versus 24.4% less likely; *p* = 0.06), and significantly more said they were less likely (rather than more likely) to request antibiotics for their child (40.4% less likely versus 24.0% more likely; *p* = 0.003).

## Discussion

In this study, we hypothesised that fear-based informational messages about AMR, intended to reduce consultations and antibiotic requests for ILI, are more likely to be effective, particularly among those with low AMR awareness, if they contain empowering information about successful self-management without antibiotics. We examined this hypothesis by developing three fear-based messages about AMR, with and without an empowerment element, and testing them in a randomised survey experiment. The aim was to inform future public campaigns intended to reduce antibiotic requests for ILI, although our findings have broader application. We found that for all the informational messages tested, there were stark differences in how respondents said the information would affect their future consultations and antibiotic requests. The magnitude of these differences depended on whether the information they were given was new to them or whether they thought antibiotics would help ILI. Those for whom the information was not new (or who did not think antibiotics would help) stated that the information was likely to make them less likely to consult or request antibiotics, regardless of which message they were given. This suggests that repeated campaigns may be important for re-educating those who think they are already well informed about appropriate antibiotic use. Crucially, however, where the information was new, *only* those given the ‘strong-fear-plus-empowerment’ message said they would be no more likely to consult *and* less likely to request antibiotics. Those given the ‘mild-fear-plus-empowerment’ message were not significantly less likely to consult or request antibiotics for ILI. This is consistent with the most recent meta-analysis on fear-appeal messages which found that in general, they are more effective when relatively high fear levels are employed [23]. There was no evidence that those given the ‘fear-only’ message were less likely to request antibiotics for ILI, and consistent with our previous work [24], they were more likely to consult.

The key study limitation is that it relies on how survey respondents claimed they would behave in response to information. Reported intentions may differ from actual behaviour. However, there is evidence from meta-analyses that intentions predict infrequent, non-habitual behaviours [25] and that changing behavioural intentions engenders behaviour change [26]. Another limitation is that only members of the online survey panel were eligible. Thus, the sample was limited to those with an interest in completing surveys, internet access, and basic computer literacy. However, the use of this panel also meant that age, gender, ethnicity, geographic region, and employment status were broadly representative of the general UK population, though the percentage with higher education (44%) was higher than the population average (27%).

Previously [24], we found that fear messages about AMR could potentially backfire among respondents poorly informed about AMR. There, those who found a ‘fear-only’ message (almost identical to version 1) ‘very/somewhat surprising’ said they were more likely to consult and to request antibiotics for ILI. Based on evidence from behavioural science [21–23], we hypothesised that the unintended response to this information may be because the original message lacked empowering information about effective self-management of ILI without antibiotics. A major study strength is that this insight from behavioural science was used, apparently successfully, to develop informational messages likely not only to avoid backfiring, but to effectively reduce antibiotic requests for ILI. With just minor changes to wording, this study also attempted, and broadly succeeded, in replicating the results reported in our previous work [24], by testing a ‘fear-only’ message (included in Figs. 1 and 2 as ‘Prior Survey’ to facilitate comparability).

To address the limitation that the respondents for whom information was ‘very/somewhat new’ varied across the three versions tested, in our exploratory analysis, we also disaggregated respondents according to beliefs about antibiotic effectiveness for ILI, rather than how new the information provided was. Using this common indicator, which did not vary across randomisations, provided similar results.

While here we developed and tested fear messages intended to improve thinking and behaviour about antibiotic use, the phenomenon we studied is widely prevalent. Meta-analyses [21–23] have identified the importance, generally, in fear campaigns of including empowering messages that the ‘call-to-action’ is relatively easy, and will be effective in mitigating the danger referred to. This is the first study to explicitly apply and test this finding in the context of fear messages about AMR. Our results strongly suggest that this general point is highly relevant to the development of public campaigns warning of the dangers of AMR, and support its importance more generally in campaigns.

We found that a ‘strong-fear-plus-empowerment’ message was likely to be effective at reducing both consultations and requests for antibiotics among adults. However, even this relatively successful version was likely to lead to increased consultation for children with ILI. The overall effect on requests for children was not consistent, with those who believed antibiotics would help their child’s ILI being more likely to take their child to a doctor, but less likely to request antibiotics if they went. It is possible that our self-care advice (plenty of fluids and sleep, or to take paracetamol) may be perceived as difficult to implement, or too generic [27], by parents of young children, and they may feel they require advice from a primary care physician about management.

Children account for a large proportion of antibiotics taken for ILI, and the factors which affect consulting and prescribing are not the same as for adults. More research is needed on developing informational messages for parents that could safely and effectively reduce unnecessary prescribing for ILI in children by reducing consultations and antibiotic requests. More broadly, future research could test fear messages which vary with regard to the nature of the empowerment content employed, incorporating respondent self-efficacy and response-efficacy elements via different forms of wording. Future research should also address whether the hypothetical future behaviour our respondents reported in response to our informational messages can be replicated in studies testing actual behaviour. In particular, it would be valuable to design a randomised trial to test the effect of the provision of informational messages, such as those developed here, on more objective measures of antibiotic use. It may also be valuable to investigate the long-term effects of such messages, as these could differ from the short-term effects. It is conceivable, for example, that repeated exposure to even the least effective message in this study could ultimately have positive long-term effects by improving AMR awareness over time and thereby making respondents more likely to respond favourably to future messages. More generally, our results emphasise the importance of testing any proposed public antibiotic-stewardship campaigns before they are rolled out to wide audiences.

## Conclusion

This study developed and tested, using a randomised design, fear-based messages about antibiotics and AMR, both with and without empowering information that influenza-like symptoms are self-limiting and can easily be self-managed, e.g. with paracetamol, rest, and fluids. Consistent with meta-analyses on the effectiveness of fear-based messages in other healthcare areas, the evidence from this study suggests that fear-based messages about antibiotics and AMR are more likely to be effective in reducing consultations and antibiotic requests, especially among those with low AMR awareness, if they include ‘empowering’ information about effective self-management without antibiotics.

## Supplementary information

**Additional file 1.** Survey instrument.

**Additional file 2: Table S1.** Respondent characteristics in Wave-1 (October/November 2016). **Table S2a.** Wave-1 (October/November 2016): How will this [AMR] information [Version 1 ‘fear-only’] affect the number of times that you/your child visit a doctor for conditions like Health State A and ask for antibiotics for you/your child? **Table S2b.** Wave-1 (October/November 2016): How will this [AMR] information [Version 2 ‘mild-fear-plus-empowerment’] affect the number of times that you/your child visit a doctor for conditions like Health State A and ask for antibiotics for you/your child? **Table S2c.** Wave-1 (October/November 2016): How will this [AMR] information [Version 3 ‘strong-fear-plus-empowerment’] affect the number of times that you/your child visit a doctor for conditions like Health State A and ask for antibiotics for you/your child? **Table S3a.** Wave-2 (March 2017): How will this [AMR] information [Version 1 ‘fear-only’] affect the number of times that you/your child visit a doctor for conditions like Health State A and ask for antibiotics for you/your child? **Table S3b.** Wave-2 (March 2017): How will this [AMR] information [Version 2 ‘mild-fear-plus-empowerment’] affect the number of times that you/your child visit a doctor for conditions like Health State A and ask for antibiotics for you/your child? **Table S3c.** Wave-2 (March 2017): How will this [AMR] information [Version 3 ‘strong-fear-plus-empowerment’] affect the number of times that you/your child visit a doctor for conditions like Health State A and ask for antibiotics for you/your child? **Table S4a.** Wave-2 (March 2017): How will this [AMR] information [Version 1 ‘fear-only’] affect the number of times that you/your child visit a doctor for conditions like Health State A and ask for antibiotics for you/your child? **Table S4b.** Wave-2 (March 2017): How will this [AMR] information [Version 2 ‘mild-fear-plus-empowerment’] affect the number of times that you/your child visit a doctor for conditions like Health State A and ask for antibiotics for you/your child? **Table S4c.** Wave-2 (March 2017):How will this [AMR] information [Version 3 ‘strong-fear-plus-empowerment’] affect the number of times that you/your child visit a doctor for conditions like Health State A and ask for antibiotics for you/your child? **Table S5a.** Wave-1 (October/November 2016): How will this [AMR] information [Version 1 ‘fear-only’] affect the number of times that you/your child visit a doctor for conditions like Health State A and ask for antibiotics for you/your child? **Table S5b.** Wave-1 (October/November 2016): How will this [AMR] information [Version 2 ‘mild-fear-plus-empowerment’] affect the number of times that you/your child visit a doctor for conditions like Health State A and ask for antibiotics for you/your child? **Table S5c.** Wave-1 (October/November 2016): How will this [AMR] information [Version 3 ‘strong-fear-plus-empowerment’] affect the number of times that you/your child visit a doctor for conditions like Health State A and ask for antibiotics for you/your child? **Table S6.** Original survey data (May/June 2015): How will this [AMR, analogous to version 1 ‘fear-only’] information affect the number of times that you/your child visit a doctor for conditions like Health State A and ask for antibiotics for you/your child? (United Kingdom, 2015).

**Additional file 3.** Overall reported response to information for respondents’ children’s ILI.

**Additional file 4.** Wording for the 3 versions of information tested in Wave-1 and Wave-2.

## Data Availability

The datasets generated and analysed during the current study are available from the corresponding author on reasonable request.

## Abbreviations

AMR: Antimicrobial resistance
DALY: Disability-adjusted life-year
ILI: Influenza-like illness
NHS: National Health Service
PHE: Public Health England
RTI: Respiratory tract infection
SSI: Survey Sampling International
UK: United Kingdom
