# Supplementary material for: Reducing expectations for antibiotics in primary care: a randomised experiment to test the response to fear-based messages about antimicrobial resistance
Source: BMC Med. 2020 Apr 23;18:110. doi: 10.1186/s12916-020-01553-6 (PMC7178623; doi:10.1186/s12916-020-01553-6)
Supplement: Supplementary file 2 — Additional file 2: Table S1. Respondent characteristics in Wave-1 (October/November 2016). Table S2a. Wave-1 (October/November 2016): How will this [AMR] information [Version 1 ‘fear-only’] affect the number of times that you/your child visit a doctor for conditions like Health State A and ask for antibiotics for you/your child? Table S2b. Wave-1 (October/November 2016): How will this [AMR] information [Version 2 ‘mild-fear-plus-empowerment’] affect the number of times that you/your child visit a doctor for conditions like Health State A and ask for antibiotics for you/your child? Table S2c. Wave-1 (October/November 2016): How will this [AMR] information [Version 3 ‘strong-fear-plus-empowerment’] affect the number of times that you/your child visit a doctor for conditions like Health State A and ask for antibiotics for you/your child? Table S3a. Wave-2 (March 2017): How will this [AMR] information [Version 1 ‘fear-only’] affect the number of times that you/your child visit a doctor for conditions like Health State A and ask for antibiotics for you/your child? Table S3b. Wave-2 (March 2017): How will this [AMR] information [Version 2 ‘mild-fear-plus-empowerment’] affect the number of times that you/your child visit a doctor for conditions like Health State A and ask for antibiotics for you/your child? Table S3c. Wave-2 (March 2017): How will this [AMR] information [Version 3 ‘strong-fear-plus-empowerment’] affect the number of times that you/your child visit a doctor for conditions like Health State A and ask for antibiotics for you/your child? Table S4a. Wave-2 (March 2017): How will this [AMR] information [Version 1 ‘fear-only’] affect the number of times that you/your child visit a doctor for conditions like Health State A and ask for antibiotics for you/your child? Table S4b. Wave-2 (March 2017): How will this [AMR] information [Version 2 ‘mild-fear-plus-empowerment’] affect the number of times that you/your child visit a doctor for conditions like Health State A [file 12916_2020_1553_MOESM2_ESM.docx]

**Additional file 2. Supplementary Tables**

**Table S1. Respondent characteristics in Wave-1 (October/November 2016)**

|  | **Version 1: (N=1,000)**  **‘Fear-only’** | | **Version 2: (N=1,500)**  **‘Mild-fear-plus-empowerment’** | | **Version 3: (N=1,500)**  **‘Strong-fear-plus-empowerment’** | |
| --- | --- | --- | --- | --- | --- | --- |
| Variable | Mean or Number | Standard Deviation or % | Mean or Number | Standard Deviation or % | Mean or Number | Standard Deviation or % |
|  |  |  |  |  |  |  |
| Age | 47·4 | 17·1 | 46·1 | 17·0 | 46·5 | 16·6 |
| Household equivalent income (£)^1^ | 21697 | 16631 | 21689 | 17346 | 22111 | 17270 |
| Own self-rated health (0-10) | 7·3 | 1·9 | 7·2 | 1·9 | 7·3 | 2·0 |
| Male^1^ | 472 | 47·2% | 719 | 48·0% | 750 | 50·0% |
| White^1^ | 918 | 92·2% | 1345 | 90·6% | 1361 | 91·0% |
| Christian^1^ | 535 | 55·0% | 721 | 49·3% | 730 | 49·8% |
| Higher education | 440 | 44·0% | 710 | 47·3% | 725 | 48·3% |
| Unemployed | 50 | 5·0% | 79 | 5·3% | 73 | 4·9% |
| Sick or disabled | 42 | 4·2% | 46 | 3·1% | 52 | 3·5% |
| Married / civil partnership / live with partner | 688 | 68·8% | 1019 | 67·9% | 1013 | 67·5% |
| Antibiotics would “definitely/probably” help ILI | 293 | 29·3% | 437 | 29·1% | 423 | 28·2% |
| Born in UK | 910 | 91·0% | 1361 | 90·7% | 1353 | 90·2% |
| ***Geographic Region*** |  |  |  |  |  |  |
| East Anglia | 90 | 9·0% | 148 | 9·9% | 135 | 9·0% |
| East Midlands | 82 | 8·2% | 101 | 6·7% | 107 | 7·1% |
| West Midlands | 92 | 9·2% | 132 | 8·8% | 130 | 8·7% |
| London | 118 | 11·8% | 220 | 14·7% | 178 | 11·9% |
| North East | 44 | 4·4% | 59 | 3·9% | 65 | 4·3% |
| North West | 100 | 10·0% | 185 | 12·3% | 165 | 11·0% |
| South East | 136 | 13·6% | 186 | 12·4% | 228 | 15·2% |
| South West | 106 | 10·6% | 118 | 7·9% | 119 | 7·9% |
| Yorkshire & Humberside | 71 | 7·1% | 132 | 8·8% | 134 | 8·9% |
| Wales | 53 | 5·3% | 64 | 4·3% | 80 | 5·3% |
| Scotland | 81 | 8·1% | 110 | 7·3% | 127 | 8·5% |
| Northern Ireland | 27 | 2·7 | 45 | 3·0 | 32 | 2·1 |
| AMR information is “very/somewhat new” | 303 | 30·3% | 357 | 23·8% | 392 | 26·1% |
| Antibiotics would “definitely/probably” help ILI | 293 | 29·3% | 437 | 29·1% | 423 | 28·2% |

Notes: 1. Version 1 denominator is 1000 except for (a) household income (N=916), sex (N=999), ethnicity (N=996), and religion (N=973) where there was a “prefer not to answer” option;

Version 2 denominator is 1500 except for (a) household income (N=1378), sex (N=1498), ethnicity (N=1484), and religion (N=1461) where there was a “prefer not to answer” option;

Version 3 denominator is 1500 except for (a) household income (N=1370), sex (N=1499), ethnicity (N=1496), and religion (N=1465) where there was a “prefer not to answer” option

**Table S2a. Wave-1 (October/November 2016): How will this [AMR] information [Version 1 ‘fear-only’] affect the number of times that you/your child visit a doctor for conditions like Health State A and ask for antibiotics for you/your child?**

|  | For oneself (adult) | | For one’s child | |
| --- | --- | --- | --- | --- |
|  | Information on AMR new | Information on AMR not new | Information on AMR new | Information on AMR not new |
| Visits to GP (N) | 303 | 697 | 146 | 241 |
| Much less likely visit | 22 (7·3%) | 93 (13·3%) | 8 (5·5%) | 18 (7·5%) |
| Less likely visit | 25 (8·3%) | 82 (11·8%) | 12 (8·2%) | 21 (8·7%) |
| No change | 122 (40·3%) | 471 (67·6%) | 45 (30·8%) | 173 (71·8%) |
| More likely visit | 78 (25·7%) | 30 (4·3%) | 48 (32·9%) | 16 (6·6%) |
| Much more likely visit | 44 (14·5%) | 10 (1·4%) | 27 (18·5%) | 5 (2·1%) |
| Don’t know | 12 (4·0%) | 11 (1·6%) | 6 (4·1%) | 8 (3·3%) |
| P (change within group)^1^ | <0·0001 | <0·0001 | <0·0001 | 0·01 |
| P (difference between groups)^2^ | <0·0001 | | <0·0001 | |
|  |  |  |  |  |
| Ask for antibiotics (N) | 303 | 697 | 146 | 241 |
| Much less likely ask | 28 (9·2%) | 175 (25·1%) | 9 (6·2%) | 32 (13·3%) |
| Less likely ask | 41 (13·5%) | 113 (16·2%) | 27 (18·5%) | 40 (16·6%) |
| No change | 114 (37·6%) | 364 (52·2%) | 54 (37·0%) | 143 (59·3%) |
| More likely ask | 65 (21·5%) | 12 (1·7%) | 34 (23·3%) | 6 (2·5%) |
| Much more likely ask | 33 (10·9%) | 7 (1·0%) | 18 (12·3%) | 3 (1·2%) |
| Don’t know | 22 (7·3%) | 26 (3·7%) | 4 (2·7%) | 17 (7·1%) |
| P (change within group) ^1^ | 0·04 | <0·0001 | 0·06 | <0·0001 |
| P (difference between groups)^2^ | <0·0001 | | <0·0001 | |

Note: p-values calculated excluding “Don’t know” category and coding a 5-point Likert scale for each variable, 1. Wilcoxon single-sample signed-rank test that the median value within each group is no change; 2 Wilcoxon rank-sum test comparing the distribution of responses in those who do and do not say AMR information is new.

**Table S2b. Wave-1 (October/November 2016): How will this [AMR] information [Version 2 ‘mild-fear-plus-empowerment’] affect the number of times that you/your child visit a doctor for conditions like Health State A and ask for antibiotics for you/your child?**

|  | For oneself (adult) | | For one’s child | |
| --- | --- | --- | --- | --- |
|  | Information on AMR new | Information on AMR not new | Information on AMR new | Information on AMR  not new |
| Visits to GP (N) | 357 | 1,143 | 223 | 386 |
| Much less likely visit | 37 (10·4%) | 332 (29·1%) | 11 (4·9%) | 64 (16·6%) |
| Less likely visit | 68 (19·1%) | 239 (20·9%) | 30 (13·5%) | 71 (18·4%) |
| No change | 85 (23·8%) | 503 (44·0%) | 73 (32·7%) | 201 (52·1%) |
| More likely visit | 104 (29·1%) | 34 (3·0%) | 67 (30·0%) | 24 (6·2%) |
| Much more likely visit | 48 (13·5%) | 13 (1·1%) | 36 (16·1%) | 15 (3·9%) |
| Don’t know | 15 (4·2%) | 22 (1·9%) | 6 (2·7%) | 11 (2·9%) |
| P (change within group)^1^ | 0·01 | <0·0001 | <0·0001 | <0·0001 |
| P (difference between groups)^2^ | <0·0001 | | <0·0001 | |
|  |  |  |  |  |
| Ask for antibiotics (N) | 357 | 1,143 | 223 | 386 |
| Much less likely ask | 53 (14·9%) | 469 (41·0%) | 24 (10·8%) | 121 (31·4%) |
| Less likely ask | 71 (19·9%) | 159 (13·9%) | 41 (18·4%) | 70 (18·1%) |
| No change | 88 (24·7%) | 450 (39·4%) | 50 (22·4%) | 165 (42·8%) |
| More likely ask | 94 (26·3%) | 23 (2·0%) | 67 (30·0%) | 8 (2·1%) |
| Much more likely ask | 35 (9·8%) | 8 (0·7%) | 31 (13·9%) | 10 (2·6%) |
| Don’t know | 16 (4·5%) | 34 (3·0%) | 10 (4·5%) | 12 (3·1%) |
| P (change within group) ^1^ | 0·69 | <0·0001 | 0·02 | <0·0001 |
| P (difference between groups)^2^ | <0·0001 | | <0·0001 | |

Note: p-values calculated excluding “Don’t know” category and coding a 5-point Likert scale for each variable, 1. Wilcoxon single-sample signed-rank test that the median value within each group is no change; 2 Wilcoxon rank-sum test comparing the distribution of responses in those who do and do not say AMR information is new.

**Table S2c. Wave-1 (October/November 2016): How will this [AMR] information [Version 3 ‘strong-fear-plus-empowerment’] affect the number of times that you/your child visit a doctor for conditions like Health State A and ask for antibiotics for you/your child?**

|  | For oneself (adult) | | For one’s child | |
| --- | --- | --- | --- | --- |
|  | Information on AMR new | Information on AMR not new | Information on AMR new | Information on AMR  not new |
| Visits to GP (N) | 392 | 1,108 | 212 | 392 |
| Much less likely visit | 52 (13·3%) | 296 (26·7%) | 13 (6·1%) | 62 (15·8%) |
| Less likely visit | 87 (22·2%) | 211 (19·0%) | 33 (15·6%) | 80 (20·4%) |
| No change | 104 (26·5%) | 533 (48·1%) | 71 (33·5%) | 217 (55·4%) |
| More likely visit | 84 (21·4%) | 31 (2·8%) | 42 (19·8%) | 12 (3·1%) |
| Much more likely visit | 57 (14·5%) | 10 (0·9%) | 48 (22·6%) | 15 (3·8%) |
| Don’t know | 8 (2·0%) | 27 (2·4%) | 5 (2·4%) | 6 (1·5%) |
| P (change within group)^1^ | 0·80 | <0·0001 | <0·0001 | <0·0001 |
| P (difference between groups)^2^ | <0·0001 | | <0·0001 | |
|  |  |  |  |  |
| Ask for antibiotics (N) | 392 | 1,108 | 212 | 392 |
| Much less likely ask | 75 (19·1%) | 440 (39·7%) | 34 (16·0%) | 111 (28·3%) |
| Less likely ask | 97 (24·7%) | 166 (15·0%) | 38 (17·9%) | 76 (19·4%) |
| No change | 97 (24·7%) | 451 (40·7%) | 49 (23·1%) | 180 (45·9%) |
| More likely ask | 75 (19·1%) | 13 (1·2%) | 50 (23·6%) | 12 (3·1%) |
| Much more likely ask | 33 (8·4%) | 7 (0·6%) | 30 (14·2%) | 3 (0·8%) |
| Don’t know | 15 (3·8%) | 31 (2·8%) | 11 (5·2%) | 10 (2·6%) |
| P (change within group) ^1^ | <0·0001 | <0·0001 | 0·78 | <0·0001 |
| P (difference between groups)^2^ | <0·0001 | | <0·0001 | |

Note: p-values calculated excluding “Don’t know” category and coding a 5-point Likert scale for each variable, 1. Wilcoxon single-sample signed-rank test that the median value within each group is no change; 2 Wilcoxon rank-sum test comparing the distribution of responses in those who do and do not say AMR information is new.

**Table S3a. Wave-2 (March 2017): How will this [AMR] information [Version 1 ‘fear-only’] affect the number of times that you/your child visit a doctor for conditions like Health State A and ask for antibiotics for you/your child?**

|  | For oneself (adult) | | For one’s child | |
| --- | --- | --- | --- | --- |
|  | Information on AMR new | Information on AMR not new | Information on AMR new | Information on AMR  not new |
| Visits to GP (N) | 285 | 715 | 134 | 274 |
| Much less likely visit | 16 (5·6%) | 109 (15·2%) | 7 (5·2%) | 25 (9·1%) |
| Less likely visit | 46 (16·1%) | 121 (16·9%) | 9 (6·7%) | 37 (13·5%) |
| No change | 105 (36·8%) | 437 (61·1%) | 46 (34·3%) | 179 (65·3%) |
| More likely visit | 69 (24·2%) | 24 (3·4%) | 39 (29·1%) | 13 (4·7%) |
| Much more likely visit | 40 (14·0%) | 8 (1·1%) | 28 (20·9%) | 13 (4·7%) |
| Don’t know | 9 (3·2%) | 16 (2·2%) | 5 (3·7%) | 7 (2·6%) |
| P (change within group)^1^ | 0·0001 | <0·0001 | <0·0001 | 0·0002 |
| P (difference between groups)^2^ | <0·0001 | | <0·0001 | |
|  |  |  |  |  |
| Ask for antibiotics (N) | 285 | 715 | 134 | 274 |
| Much less likely ask | 30 (10·5%) | 195 (27·3%) | 12 (9·0%) | 54 (19·7%) |
| Less likely ask | 55 (19·3%) | 143 (20·0%) | 18 (13·4%) | 57 (20·8%) |
| No change | 95 (33·3%) | 348 (48·7%) | 50 (37·3%) | 145 (52·9%) |
| More likely ask | 73 (25·6%) | 8 (1·1%) | 29 (21·6%) | 5 (1·8%) |
| Much more likely ask | 18 (6·3%) | 2 (0·3%) | 19 (14·2%) | 3 (1·1%) |
| Don’t know | 14 (4·9%) | 19 (2·7%) | 6 (4·5%) | 10 (3·7%) |
| P (change within group) ^1^ | 0·97 | <0·0001 | 0·047 | <0·0001 |
| P (difference between groups)^2^ | <0·0001 | | <0·0001 | |

Note: p-values calculated excluding “Don’t know” category and coding a 5-point Likert scale for each variable, 1. Wilcoxon single-sample signed-rank test that the median value within each group is no change; 2 Wilcoxon rank-sum test comparing the distribution of responses in those who do and do not say AMR information is new.

**Table S3b. Wave-2 (March 2017): How will this [AMR] information [Version 2 ‘mild-fear-plus-empowerment’] affect the number of times that you/your child visit a doctor for conditions like Health State A and ask for antibiotics for you/your child?**

|  | For oneself (adult) | | For one’s child | |
| --- | --- | --- | --- | --- |
|  | Information on AMR new | Information on AMR not new | Information on AMR new | Information on AMR  not new |
| Visits to GP (N) | 336 | 1,164 | 169 | 418 |
| Much less likely visit | 41 (12·2%) | 333 (28·6%) | 5 (3·0%) | 66 (15·8%) |
| Less likely visit | 56 (16·7%) | 247 (21·2%) | 23 (13·6%) | 81 (19·4%) |
| No change | 96 (28·6%) | 531 (45·6%) | 46 (27·2%) | 216 (51·7%) |
| More likely visit | 82 (24·4%) | 20 (1·7%) | 53 (31·4%) | 23 (5·5%) |
| Much more likely visit | 47 (14·0%) | 5 (0·4%) | 38 (22·5%) | 19 (4·6%) |
| Don’t know | 14 (4·2%) | 28 (2·4%) | 4 (2·4%) | 13 (3·1%) |
| P (change within group)^1^ | 0·07 | <0·0001 | <0·0001 | <0·0001 |
| P (difference between groups)^2^ | <0·0001 | | <0·0001 | |
|  |  |  |  |  |
| Ask for antibiotics (N) | 336 | 1,164 | 169 | 418 |
| Much less likely ask | 61 (18·2%) | 473 (40·6%) | 16 (9·5%) | 111 (26·6%) |
| Less likely ask | 55 (16·4%) | 198 (17·0%) | 27 (16·0%) | 88 (21·1%) |
| No change | 101 (30·1%) | 442 (38·0%) | 49 (29·0%) | 185 (44·3%) |
| More likely ask | 62 (18·5%) | 16 (1·4%) | 41 (24·3%) | 11 (2·6%) |
| Much more likely ask | 41 (12·2%) | 4 (0·3%) | 25 (14·8%) | 9 (2·2%) |
| Don’t know | 16 (4·8%) | 31 (2·7%) | 11 (6·5%) | 14 (3·4%) |
| P (change within group) ^1^ | 0·19 | <0·0001 | 0·031 | <0·0001 |
| P (difference between groups)^2^ | <0·0001 | | <0·0001 | |

Note: p-values calculated excluding “Don’t know” category and coding a 5-point Likert scale for each variable, 1. Wilcoxon single-sample signed-rank test that the median value within each group is no change; 2 Wilcoxon rank-sum test comparing the distribution of responses in those who do and do not say AMR information is new.

**Table S3c. Wave-2 (March 2017): How will this [AMR] information [Version 3 ‘strong-fear-plus-empowerment’] affect the number of times that you/your child visit a doctor for conditions like Health State A and ask for antibiotics for you/your child?**

|  | For oneself (adult) | | For one’s child | |
| --- | --- | --- | --- | --- |
|  | Information on AMR new | Information on AMR not new | Information on AMR new | Information on AMR  not new |
| Visits to GP (N) | 388 | 1,112 | 193 | 412 |
| Much less likely visit | 49 (12·6%) | 283 (25·5%) | 9 (4·7%) | 71 (17·2%) |
| Less likely visit | 89 (22·9%) | 271 (24·4%) | 30 (15·5%) | 91 (22·1%) |
| No change | 104 (26·8%) | 509 (45·8%) | 66 (34·2%) | 218 (52·9%) |
| More likely visit | 90 (23·2%) | 25 (2·3%) | 52 (26·9%) | 13 (3·2%) |
| Much more likely visit | 46 (11·9%) | 9 (0·8%) | 31 (16·1%) | 9 (2·2%) |
| Don’t know | 10 (2·6%) | 15 (1·4%) | 5 (2·6%) | 10 (2·4%) |
| P (change within group)^1^ | 0·85 | <0·0001 | <0·0001 | <0·0001 |
| P (difference between groups)^2^ | <0·0001 | | <0·0001 | |
|  |  |  |  |  |
| Ask for antibiotics (N) | 388 | 1,112 | 193 | 412 |
| Much less likely ask | 89 (22·9%) | 462 (41·6%) | 28 (14·5%) | 128 (31·1%) |
| Less likely ask | 93 (24·0%) | 177 (15·9%) | 45 (23·3%) | 88 (21·4%) |
| No change | 100 (25·8%) | 424 (38·1%) | 54 (28·0%) | 172 (41·8%) |
| More likely ask | 69 (17·8%) | 12 (1·1%) | 36 (18·7%) | 9 (2·2%) |
| Much more likely ask | 26 (6·7%) | 6 (0·5%) | 24 (12·4%) | 3 (0·7%) |
| Don’t know | 11 (2·8%) | 31 (2·8%) | 6 (3·1%) | 12 (2·9%) |
| P (change within group) ^1^ | <0·0001 | <0·0001 | 0·30 | <0·0001 |
| P (difference between groups)^2^ | <0·0001 | | <0·0001 | |

Note: p-values calculated excluding “Don’t know” category and coding a 5-point Likert scale for each variable, 1. Wilcoxon single-sample signed-rank test that the median value within each group is no change; 2 Wilcoxon rank-sum test comparing the distribution of responses in those who do and do not say AMR information is new.

**Table S4a. Wave-2 (March 2017): How will this [AMR] information [Version 1 ‘fear-only’] affect the number of times that you/your child visit a doctor for conditions like Health State A and ask for antibiotics for you/your child?**

|  | For oneself (adult) | | For one’s child | |
| --- | --- | --- | --- | --- |
|  | Think antibiotics would help ILI | Don’t think antibiotics would help ILI or don’t know | Think antibiotics would help child with ILI | Don’t think antibiotics would help child with ILI or don’t know |
| Visits to GP (N) | 279 | 721 | 183 | 225 |
| Much less likely visit | 9 (3·2%) | 116 (16·1%) | 6 (3·3%) | 26 (11·6%) |
| Less likely visit | 36 (12·9%) | 131 (18·2%) | 11 (6·0%) | 35 (15·6%) |
| No change | 131 (47·0%) | 411 (57·0%) | 84 (45·9%) | 141 (62·7%) |
| More likely visit | 58 (20·8%) | 35 (4·9%) | 43 (23·5%) | 9 (4·0%) |
| Much more likely visit | 39 (14·0%) | 9 (1·3%) | 35 (19·1%) | 6 (2·7%) |
| Don’t know | 6 (2·2%) | 19 (2·6%) | 4 (2·2%) | 8 (3·6%) |
| P (change within group)^1^ | <0·0001 | <0·0001 | <0·0001 | <0·0001 |
| P (difference between groups)^2^ | <0·0001 | | <0·0001 | |
|  |  |  |  |  |
| Ask for antibiotics (N) | 279 | 721 | 183 | 225 |
| Much less likely ask | 18 (6·5%) | 207 (28·7%) | 11 (6·0%) | 55 (24·4%) |
| Less likely ask | 56 (20·1%) | 142 (19·7%) | 30 (16·4%) | 45 (20·0%) |
| No change | 118 (42·3%) | 325 (45·1%) | 87 (47·5%) | 108 (48·0%) |
| More likely ask | 60 (21·5%) | 21 (2·9%) | 31 (16·9%) | 3 (1·3%) |
| Much more likely ask | 17 (6·1%) | 3 (0·4%) | 19 (10·4%) | 3 (1·3%) |
| Don’t know | 10 (3·6%) | 23 (3·2%) | 5 (2·7%) | 11 (4·9%) |
| P (change within group) ^1^ | 0·85 | <0·0001 | 0·27 | <0·0001 |
| P (difference between groups)^2^ | <0·0001 | | <0·0001 | |

Note: p-values calculated excluding “Don’t know” category and coding a 5-point Likert scale for each variable, 1. Wilcoxon single-sample signed-rank test that the median value within each group is no change; 2 Wilcoxon rank-sum test comparing the distribution of responses in those who do and do not think antibiotics would help ILI.

**Table S4b. Wave-2 (March 2017): How will this [AMR] information [Version 2 ‘mild-fear-plus-empowerment’] affect the number of times that you/your child visit a doctor for conditions like Health State A and ask for antibiotics for you/your child?**

|  | For oneself (adult) | | For one’s child | |
| --- | --- | --- | --- | --- |
|  | Think antibiotics would help ILI | Don’t think antibiotics would help ILI or don’t know | Think antibiotics would help child with ILI | Don’t think antibiotics would help child with ILI or don’t know |
| Visits to GP (N) | 415 | 1,085 | 255 | 332 |
| Much less likely visit | 44 (10·6%) | 316 (29·1%) | 11 (4·3%) | 60 (18·1%) |
| Less likely visit | 70 (16·9%) | 212 (19·5%) | 28 (11·0%) | 76 (22·9%) |
| No change | 143 (34·5%) | 484 (44·6%) | 105 (41·2%) | 157 (47·3%) |
| More likely visit | 91 (21·9%) | 32 (3·0%) | 57 (22·4%) | 19 (5·7%) |
| Much more likely visit | 58 (14·0%) | 8 (0·7%) | 50 (19·6%) | 7 (2·1%) |
| Don’t know | 9 (2·2%) | 33 (3·0%) | 4 (1·6%) | 13 (3·9%) |
| P (change within group)^1^ | 0·03 | <0·0001 | <0·0001 | <0·0001 |
| P (difference between groups)^2^ | <0·0001 | | <0·0001 | |
|  |  |  |  |  |
| Ask for antibiotics (N) | 415 | 1,085 | 255 | 332 |
| Much less likely ask | 88 (21·2%) | 446 (41·1%) | 32 (12·6%) | 95 (28·6%) |
| Less likely ask | 85 (20·5%) | 168 (15·5%) | 39 (15·3%) | 76 (22·9%) |
| No change | 135 (32·5%) | 408 (37·6%) | 98 (38·4%) | 136 (41·0%) |
| More likely ask | 56 (13·5%) | 22 (2·0%) | 46 (18·0%) | 6 (1·8%) |
| Much more likely ask | 41 (9·9%) | 4 (0·4%) | 33 (12·9%) | 1 (0·3%) |
| Don’t know | 10 (2·4%) | 37 (3·4%) | 7 (2·8%) | 18 (5·4%) |
| P (change within group) ^1^ | <0·0001 | <0·0001 | 0·58 | <0·0001 |
| P (difference between groups)^2^ | <0·0001 | | <0·0001 | |

Note: p-values calculated excluding “Don’t know” category and coding a 5-point Likert scale for each variable, 1. Wilcoxon single-sample signed-rank test that the median value within each group is no change; 2 Wilcoxon rank-sum test comparing the distribution of responses in those who do and do not think antibiotics would help ILI.

**Table S4c. Wave-2 (March 2017):How will this [AMR] information [Version 3 ‘strong-fear-plus-empowerment’] affect the number of times that you/your child visit a doctor for conditions like Health State A and ask for antibiotics for you/your child?**

|  | For oneself (adult) | | For one’s child | |
| --- | --- | --- | --- | --- |
|  | Think antibiotics would help ILI | Don’t think antibiotics would help ILI or don’t know | Think antibiotics would help child with ILI | Don’t think antibiotics would help child with ILI or don’t know |
| Visits to GP (N) | 418 | 1,082 | 250 | 355 |
| Much less likely visit | 49 (11·7%) | 283 (26·2%) | 15 (6·0%) | 65 (18·3%) |
| Less likely visit | 110 (26·3%) | 250 (23·1%) | 46 (18·4%) | 75 (21·1%) |
| No change | 133 (31·8%) | 480 (44·4%) | 106 (42·4%) | 178 (50·1%) |
| More likely visit | 82 (19·6%) | 33 (3·1%) | 47 (18·8%) | 18 (5·1%) |
| Much more likely visit | 39 (9·3%) | 16 (1·5%) | 32 (12·8%) | 8 (2·3%) |
| Don’t know | 5 (1·2%) | 20 (1·9%) | 4 (1·6%) | 11 (3·1%) |
| P (change within group)^1^ | 0·03 | <0·0001 | 0·06 | <0·0001 |
| P (difference between groups)^2^ | <0·0001 | | <0·0001 | |
|  |  |  |  |  |
| Ask for antibiotics (N) | 418 | 1,082 | 250 | 355 |
| Much less likely ask | 89 (21·3%) | 462 (42·7%) | 36 (14·4%) | 120 (33·8%) |
| Less likely ask | 108 (25·8%) | 162 (15·0%) | 65 (26·0%) | 68 (19·2%) |
| No change | 121 (29·0%) | 403 (37·3%) | 84 (33·6%) | 142 (40·0%) |
| More likely ask | 65 (15·6%) | 16 (1·5%) | 36 (14·4%) | 18 (4·3%) |
| Much more likely ask | 26 (6·2%) | 6 (0·6%) | 24 (9·6%) | 9 (2·5%) |
| Don’t know | 9 (2·2%) | 33 (3·1%) | 5 (2·0%) | 3 (0·9%) |
| P (change within group) ^1^ | <0·0001 | <0·0001 | 0·003 | <0·0001 |
| P (difference between groups)^2^ | <0·0001 | | <0·0001 | |

Note: p-values calculated excluding “Don’t know” category and coding a 5-point Likert scale for each variable, 1. Wilcoxon single-sample signed-rank test that the median value within each group is no change; 2 Wilcoxon rank-sum test comparing the distribution of responses in those who do and do not think antibiotics would help ILI.

**Table S5a. Wave-1 (October/November 2016): How will this [AMR] information [Version 1 ‘fear-only’] affect the number of times that you/your child visit a doctor for conditions like Health State A and ask for antibiotics for you/your child?**

|  | For oneself (adult) | | For one’s child | |
| --- | --- | --- | --- | --- |
|  | Think antibiotics would help ILI | Don’t think antibiotics would help ILI or don’t know | Think antibiotics would help child with ILI | Don’t think antibiotics would help child with ILI or don’t know |
| Visits to GP (N) | 293 | 707 | 184 | 203 |
| Much less likely visit | 9 (3·1%) | 106 (15·0%) | 6 (3·3%) | 20 (9·9%) |
| Less likely visit | 23 (7·9%) | 84 (11·9%) | 15 (8·2%) | 18 (8·9%) |
| No change | 135 (46·1%) | 458 (64·8%) | 77 (41·9%) | 141 (69·5%) |
| More likely visit | 75 (25·6%) | 33 (4·7%) | 50 (27·2%) | 14 (6·9%) |
| Much more likely visit | 44 (15·0%) | 10 (1·4%) | 28 (15·2%) | 4 (2·0%) |
| Don’t know | 7 (2·4%) | 16 (2·3%) | 8 (4·4%) | 6 (3·0%) |
| P (change within group)^1^ | <0·0001 | <0·0001 | <0·0001 | 0·005 |
| P (difference between groups)^2^ | <0·0001 | | <0·0001 | |
|  |  |  |  |  |
| Ask for antibiotics (N) | 293 | 707 | 184 | 203 |
| Much less likely ask | 13 (4·4%) | 190 (26·9%) | 9 (4·9%) | 32 (15·8%) |
| Less likely ask | 47 (16·0%) | 107 (15·1%) | 32 (17·4%) | 35 (17·2%) |
| No change | 133 (45·4%) | 345 (48·8%) | 81 (44·0%) | 116 (57·1%) |
| More likely ask | 56 (19·1%) | 21 (3·0%) | 36 (19·6%) | 4 (2·0%) |
| Much more likely ask | 31 (10·6%) | 9 (1·3%) | 18 (9·8%) | 3 (1·5%) |
| Don’t know | 13 (4·4%) | 35 (5·0%) | 8 (4·4%) | 13 (6·4%) |
| P (change within group) ^1^ | 0·01 | <0·0001 | 0·13 | <0·0001 |
| P (difference between groups)^2^ | <0·0001 | | <0·0001 | |

Note: p-values calculated excluding “Don’t know” category and coding a 5-point Likert scale for each variable, 1. Wilcoxon single-sample signed-rank test that the median value within each group is no change; 2 Wilcoxon rank-sum test comparing the distribution of responses in those who do and do not think antibiotics would help ILI.

**Table S5b. Wave-1 (October/November 2016): How will this [AMR] information [Version 2 ‘mild-fear-plus-empowerment’] affect the number of times that you/your child visit a doctor for conditions like Health State A and ask for antibiotics for you/your child?**

|  | For oneself (adult) | | For one’s child | |
| --- | --- | --- | --- | --- |
|  | Think antibiotics would help ILI | Don’t think antibiotics would help ILI or don’t know | Think antibiotics would help child with ILI | Don’t think antibiotics would help child with ILI or don’t know |
| Visits to GP (N) | 437 | 1,063 | 278 | 331 |
| Much less likely visit | 53 (12·1%) | 316 (29·7%) | 11 (4·0%) | 64 (19·3%) |
| Less likely visit | 92 (21·1%) | 215 (20·2%) | 42 (15·1%) | 59 (17·8%) |
| No change | 131 (30·0%) | 457 (43·0%) | 111 (39·9%) | 163 (49·2%) |
| More likely visit | 95 (21·7%) | 43 (4·1%) | 62 (22·3%) | 29 (8·8%) |
| Much more likely visit | 52 (11·9%) | 9 (0·9%) | 46 (16·6%) | 5 (1·5%) |
| Don’t know | 14 (3·2%) | 23 (2·2%) | 6 (2·2%) | 11 (3·3%) |
| P (change within group)^1^ | 0·95 | <0·0001 | <0·0001 | <0·0001 |
| P (difference between groups)^2^ | <0·0001 | | <0·0001 | |
|  |  |  |  |  |
| Ask for antibiotics (N) | 437 | 1,063 | 278 | 331 |
| Much less likely ask | 74 (16·9%) | 448 (42·1%) | 32 (11·5%) | 113 (34·1%) |
| Less likely ask | 108 (24·7%) | 122 (11·5%) | 51 (18·4%) | 60 (18·1%) |
| No change | 122 (27·9%) | 416 (39·1%) | 89 (32·0%) | 126 (38·1%) |
| More likely ask | 81 (18·5%) | 36 (3·4%) | 62 (22·3%) | 13 (3·9%) |
| Much more likely ask | 37 (8·5%) | 6 (0·6%) | 37 (13·3%) | 4 (1·2%) |
| Don’t know | 15 (3·4%) | 35 (3·3%) | 7 (2·5%) | 15 (4·5%) |
| P (change within group) ^1^ | 0·0001 | <0·0001 | 0·27 | <0·0001 |
| P (difference between groups)^2^ | <0·0001 | | <0·0001 | |

Note: p-values calculated excluding “Don’t know” category and coding a 5-point Likert scale for each variable, 1. Wilcoxon single-sample signed-rank test that the median value within each group is no change; 2 Wilcoxon rank-sum test comparing the distribution of responses in those who do and do not think antibiotics would help ILI.

**Table S5c. Wave-1 (October/November 2016): How will this [AMR] information [Version 3 ‘strong-fear-plus-empowerment’] affect the number of times that you/your child visit a doctor for conditions like Health State A and ask for antibiotics for you/your child?**

|  | For oneself (adult) | | For one’s child | |
| --- | --- | --- | --- | --- |
|  | Think antibiotics would help ILI | Don’t think antibiotics would help ILI or don’t know | Think antibiotics would help child with ILI | Don’t think antibiotics would help child with ILI or don’t know |
| Visits to GP (N) | 423 | 1,077 | 278 | 326 |
| Much less likely visit | 49 (11·6%) | 299 (27·8%) | 15 (5·4%) | 60 (18·4%) |
| Less likely visit | 103 (24·4%) | 195 (18·1%) | 49 (17·6%) | 64 (19·6%) |
| No change | 132 (31·2%) | 505 (46·9%) | 111 (39·9%) | 177 (54·3%) |
| More likely visit | 79 (18·7%) | 36 (3·3%) | 49 (17·6%) | 5 (1·5%) |
| Much more likely visit | 55 (13·0%) | 12 (1·1%) | 52 (18·7%) | 11 (3·4%) |
| Don’t know | 5 (1·2%) | 30 (2·8%) | 2 (0·7%) | 9 (2·8%) |
| P (change within group)^1^ | 0·51 | <0·0001 | 0·0004 | <0·0001 |
| P (difference between groups)^2^ | <0·0001 | | <0·0001 | |
|  |  |  |  |  |
| Ask for antibiotics (N) | 423 | 1,077 | 278 | 326 |
| Much less likely ask | 74 (17·5%) | 441 (41·0%) | 39 (14·0%) | 106 (32·5%) |
| Less likely ask | 116 (27·4%) | 147 (13·7%) | 56 (20·1%) | 58 (17·8%) |
| No change | 121 (28·6%) | 427 (39·7%) | 90 (32·4%) | 139 (42·6%) |
| More likely ask | 68 (16·1%) | 20 (1·9%) | 56 (20·1%) | 6 (1·8%) |
| Much more likely ask | 34 (8·0%) | 6 (0·6%) | 30 (10·8%) | 3 (0·9%) |
| Don’t know | 10 (2·4%) | 36 (3·3%) | 7 (2·5%) | 14 (4·3%) |
| P (change within group) ^1^ | <0·0001 | <0·0001 | 0·39 | <0·0001 |
| P (difference between groups)^2^ | <0·0001 | | <0·0001 | |

Note: p-values calculated excluding “Don’t know” category and coding a 5-point Likert scale for each variable, 1. Wilcoxon single-sample signed-rank test that the median value within each group is no change; 2 Wilcoxon rank-sum test comparing the distribution of responses in those who do and do not think antibiotics would help ILI.

**Table S6. Original survey data (May/June 2015): How will this [AMR, analogous to version 1 ‘fear-only’] information affect the number of times that you/your child visit a doctor for conditions like Health State A and ask for antibiotics for you/your child? (United Kingdom, 2015)**

|  | For oneself (adult) | | For one’s child | |
| --- | --- | --- | --- | --- |
|  | Think antibiotics would help ILI | Don’t think antibiotics would help ILI or don’t know | Think antibiotics would help child with ILI | Don’t think antibiotics would help child with ILI or don’t know |
| Visits to GP (N) | 762 | 1,302 | 430 | 386 |
| Definitely visit less | 18 (2·4%) | 142 (10·9%) | 8 (1·9%) | 18 (4·7%) |
| Probably visit less | 81 (10·6%) | 124 (9·5%) | 35 (8·1%) | 44 (11·4%) |
| No change | 413 (51·2%) | 895 (68·7%) | 220 (51·2%) | 278 (72·0%) |
| Probably visit more | 155 (20·3%) | 68 (5·2%) | 100 (23·3%) | 16 (4·2%) |
| Definitely visit more | 77 (10·1%) | 28 (2·2%) | 59 (13·7%) | 11 (2·9%) |
| Don’t know | 18 (2·4%) | 45 (3·5%) | 8 (1·9%) | 19 (4·9%) |
| P (change within group)a | <0·0001 | <0·0001 | <0·0001 | 0·0003 |
| P (difference between groups)b | <0·0001 | | <0·0001 | |
|  |  |  |  |  |
| Ask for antibiotics (N) | 762 | 1,302 | 430 | 386 |
| Definitely ask less | 31 (4·1%) | 203 (15·6%) | 16 (3·7%) | 33 (8·6%) |
| Probably ask less | 100 (13·1%) | 154 (11·8%) | 52 (12·1%) | 59 (15·3%) |
| No change | 383 (50·3%) | 817 (62·8%) | 195 (45·4%) | 249 (64·5%) |
| Probably ask more | 152 (20·0%) | 50 (3·8%) | 108 (25·1%) | 10 (2·6%) |
| Definitely ask more | 80 (10·5%) | 21 (1·6%) | 50 (11·6%) | 10 (2·6%) |
| Don’t know | 16 (2·1%) | 57 (4·4%) | 9 (2·1%) | 25 (6·5%) |
| P (change within group) a | <0·0001 | <0·0001 | <0·0001 | <0·0001 |
| P (difference between groups)b | <0·0001 | | <0·0001 | |

Note: p-values calculated excluding “Don’t know” category and coding a 5-point Likert scale for each variable, a. Wilcoxon single-sample signed-rank test that the median value within each group is no change; b. Wilcoxon rank-sum test comparing the distribution of responses in those who do and do not think antibiotics would help ILI.
