# Supplementary material for: Reducing expectations for antibiotics in primary care: a randomised experiment to test the response to fear-based messages about antimicrobial resistance
Source: BMC Med. 2020 Apr 23;18:110. doi: 10.1186/s12916-020-01553-6 (PMC7178623; doi:10.1186/s12916-020-01553-6)
Supplement: Supplementary file 3 — Additional file 3. Overall reported response to information for respondents’ children’s ILI. [file 12916_2020_1553_MOESM3_ESM.docx]

**Additional file 3. Overall reported response to information for respondents’ children’s ILI.**

Among those with children, 78/408 (19*·*1%), 175/587 (29*·*8%), and 201/605 (33*·*2%) of parents randomised to ‘fear-only’, ‘mild-fear-plus-empowerment’, and ‘strong-fear-plus-empowerment’ respectively reported that they would be ‘less/much-less likely’ to visit a doctor for their child (p<0*·*0001, Additional file 2: Table S3), while 93/408 (22*·*8%), 133/587 (22*·*7%), and 105/605 (17*·*4%) reported that they would be ‘more/much more-likely’ to visit a doctor for their child (p=0*·*07, Additional file 2: Table S3). In the event that they were to visit a doctor for these symptoms, 141/408 (34*·*6%), 242/587 (41*·*2%), and 289/605 (47*·*8%) reported that they would be ‘less/much less-likely’ to ask for antibiotics for their child (p=0*·*002, Additional file 2: Table S3), while 56/408 (13*·*7%), 86/587 (14*·*7%), and 72/605 (11*·*9%) respectively said they would be ‘more/much more-likely’ to ask for antibiotics for their child (p=0*·*48).

Response to information for respondents’ children’s ILI: information not “very/somewhat new”

Among parents for whom the information was not “very/somewhat new”, significantly more respondents said they would be less likely (rather than more likely) to consult/request antibiotics for their child’s ILI in response to each message (p<0·001, Figure 2(C/D))). Similarly, among those who did not think antibiotics would help their child’s ILI, significantly more respondents said they would be less likely (rather than more likely) to consult/request antibiotics for their child in response to each messages (p<0·0001; Figure 3(C/D)).

Response to information for respondents’ children’s ILI: information “very/somewhat new”

Among parents exposed to the ‘fear-only’ information, and for whom the information was “very/somewhat new”, significantly more said they were more likely (rather than less likely) to consult for their child’s ILI (50·0% more likely versus 11·9% less likely; p<0·0001) and to request antibiotics (35·8% more likely versus 22·4% less likely; p=0·05) (Figure 2(C/D)). Similarly, among parents given this message who believed antibiotics would “definitely/probably” help their child, significantly more said they were more likely (rather than less likely) to consult for their child’s ILI (42·6% more likely versus 9·3% less likely; p<0·0001) (Figure 3(C/D)). In this group, however, there was no evidence of differences between the proportions who said they would be more likely (versus less likely) to request antibiotics (p=0·27).

Among parents receiving the ‘mild-fear-plus-empowerment’ message, and for whom the information was “very/somewhat new”, significantly more said they were more likely (rather than less likely) to consult for their child’s ILI (53·8% more likely versus 16·6% less likely; p<0·0001). They were also more likely (versus less likely) to request antibiotics for their child (39·1% more likely versus 25·4% less likely; p=0·03). Similarly, among parents given this message who thought antibiotics would “definitely/probably” help, significantly more said they were more likely (rather than less likely) to consult for their child’s ILI (42·0% more likely versus 15·3% less likely; p<0·0001). In this group, there was no evidence of difference between the numbers who said they would be more likely (versus less likely) to request antibiotics for their child (p=0·58).

Among those parents given the ‘strong-fear-plus-empowerment’ message, and for whom the information was “very/somewhat new”, significantly more said they were more likely (rather than less likely) to consult for their child’s ILI (43·0% more likely versus 20·2% less likely; p<0·0001). However, in contrast to the other two randomised messages, they were not more likely (versus less likely) to request antibiotics for their child (31·1% more likely versus 37·8% less likely; p=0·30). Moreover, parents given this message who thought antibiotics would “definitely/probably” help were not significantly more likely (versus less likely) to consult for their child’s ILI (31·6% more likely versus 24·4% less likely; p=0·06), and significantly more said they were less likely (rather than more likely) to request antibiotics for their child (40·4% less likely versus 24·0% more likely; p=0·003).
