# Supplementary material for: Reducing expectations for antibiotics in primary care: a randomised experiment to test the response to fear-based messages about antimicrobial resistance
Source: BMC Med. 2020 Apr 23;18:110. doi: 10.1186/s12916-020-01553-6 (PMC7178623; doi:10.1186/s12916-020-01553-6)
Supplement: Supplementary file 4 — Additional file 4. Wording for the 3 versions of information tested in Wave-1 and Wave-2. [file 12916_2020_1553_MOESM4_ESM.docx]

**Additional file 4. Wording for the 3 versions of information tested in Wave-1 and Wave-2.**

**Version 1***: Antibiotic resistance happens when an antibiotic no longer kills or controls growing bacteria. It is an increasingly serious threat to public health. Without antibiotics that work well, many routine treatments will become increasingly dangerous. Setting broken bones, and even basic operations, rely on access to antibiotics that work. Antibiotic resistance is believed to be caused by unnecessary use of antibiotics, and inappropriate use, such as not taking them as prescribed, skipping doses, or saving them for later use.

**Version 2**: Most people get cold or flu symptoms every year, and these usually get better on their own. Temperatures sometimes last for days, while coughs can last for weeks, and antibiotics generally don’t help. Antibiotics should not be taken for cold and flu symptoms. Taking antibiotics when they are not needed encourages bacteria to become resistant. This means antibiotics may not work for future serious illnesses that can only be cured by antibiotics. Most cold and flu symptoms are best treated at home by taking paracetamol or ibuprofen, and getting plenty of fluids and sleep.

**Version 3**: Most people get cold or flu symptoms every year, and these usually get better on their own. Temperatures sometimes last for days, while coughs can last for weeks, and antibiotics generally don’t help. Antibiotics should not be taken for cold and flu symptoms. Taking antibiotics encourages bacteria to become resistant. Some killer diseases are already resistant to several antibiotics. Antibiotic resistance is an increasingly serious threat to everyone’s health. Soon we will not be able to find antibiotics that can cure serious illnesses. Even worse, without antibiotics that work, even minor injuries and routine operations will become increasingly dangerous. You can also pass on resistant bugs to people you care about. Most cold and flu symptoms are best treated at home by taking paracetamol or ibuprofen, and getting plenty of fluids and sleep.

* Version 1 is based on original information wording in the previous survey [24], but with minor edits to improve readability. Exact wording in Survey 1 was as below:

**Original wording:** Antibiotic resistance occurs when an antibiotic loses its ability to effectively control or kill growing bacteria. It is an increasingly serious threat to public health. Without effective antibiotics, many routine treatments will become increasingly dangerous. Setting broken bones, and even basic operations, rely on access to antibiotics that work. Antibiotic resistance is believed to be caused by unnecessary use of antibiotics, and inappropriate use, such as not taking them as prescribed, skipping doses, or saving them for later use.
